# Tailoring seed oil composition in the real world: optimising omega-3 long chain polyunsaturated fatty acid accumulation in transgenic *Camelina sativa*

**DOI:** 10.1038/s41598-017-06838-0

**Published:** 2017-07-26

**Authors:** Sarah Usher, Lihua Han, Richard P. Haslam, Louise V. Michaelson, Drew Sturtevant, Mina Aziz, Kent D. Chapman, Olga Sayanova, Johnathan A. Napier

**Affiliations:** 1 Department of Plant Sciences, Rothamsted Research, Harpenden, Herts AL5 2JQ UK; 2 University of North Texas, BioDiscovery Institute, Denton, TX 76203 USA

## Abstract

There is considerable interest in the *de novo* production of omega-3 long chain polyunsaturated fatty acids such as eicosapentaenoic acid (EPA) and docosahexaenoic acid (DHA), not least of all given the importance of these fatty acids in both aquaculture and human nutrition. Previously we have demonstrated the feasibility of using metabolic engineering in transgenic plants (*Camelina sativa*) to modify the seed oil composition to now include EPA and/or DHA. In this study, we further tailored the seed oil profile to reduce the omega-6 content, and evaluated the performance of such GM plants under field conditions (i.e. environmental releases), in terms of agronomic performance and also the lipidomic profile of seed oil. We used MALDI- mass spectrometry imaging to identify discrete tissue-types in the seed in which these non-native fatty acids preferentially accumulated. Collectively, these data provide new insights into the complexity of plant lipid metabolism and the challenges associated with predictive manipulation of these pathways. However, this study identified the likely dispensable nature of a Δ12-desturase activity in our omega-3 metabolic engineering rationales for Camelina.

## Introduction

There is considerable interest in the production of novel sources of omega-3 (ω3) long chain polyunsaturated fatty acids (LC-PUFAs), which are known to have a proven role in human health (specifically in reducing our risk of cardiovascular disease and related metabolic pathologies)^1^. The main source of omega-3 LC-PUFAs are oceanically-derived fish oils, though the primary synthesis of these fatty acids is carried out not by fish, but by marine microbes (such as microalgae, diatoms and protists) at the base of the aquatic food webs^2^. Growing demand for omega-3 LC-PUFAs, in particular from the aquaculture sector, has placed considerable pressure on the wild “reduction” fisheries from which much of these fish oils are harvested^3^. Simultaneously, the global aquaculture industry has continued to expand^4^, driven in turn by the growing human population. Collectively, these factors now challenge the assumption that farmed fish species such as salmon contain significant levels of the health beneficial omega-3 LC-PUFAs such as eicosapentaenoic acid (EPA; 20:5Δ 5,8,11,14,17) and docosahexanoic acid (DHA; 22:6Δ ^4,7,10,13,16,19^), and there is emerging evidence that farmed fish now contain significantly less of these fatty acids compared with ten years ago^5, 6^. This has great relevance in ensuring good human nutrition through maximising the availability of these healthy fatty acids, not least of all in view of the relentless global rise in cardiovascular and metabolic diseases and the burden these pathologies impose on public health systems﻿^7^. We and others have previously demonstrated the possibility of using transgenic plants to make a *de novo* terrestrial source of omega-3 LC-PUFAs such EPA and DHA^8–11^. Such a source would represent a potentially more sustainable, less environmentally-intrusive production route, and also avoid some of the other well-known issues associated with oceanically-sourced fish oils, such as contamination with heavy metals, dioxins and PCBs^3^. Moreover, by harnessing the power of agriculture, in terms of scalability, pre-existing infrastructure and know-how, significant amounts of plant-derived omega-3 “fish oils” could be produced to help replace or augment those being sourced from the oceans^2^. For all of these reasons, we have developed a transgenic oilseed platform around the boutique crop *Camelina sativa*. Camelina, a member of the Brassicacae, is an established oilseed crop grown in both Europe and North America, for a range of applications including food and feed use. Previously we transitioned our proof-of-concept work in the model system Arabidopsis^9, 12^ into this crop, and demonstrated that it is possible to direct the synthesis of EPA and DHA in Camelina seed oil to the same levels observed in *bona fide* fish oils^13, 14^. Subsequently, we have also shown the utility of these novel oils as aquafeed ingredient (as a replacement for fish oils)^15–18^. We have also recently reported the first environmental release and field evaluation of one of these first prototypes^19^.

Building on previous data which indicated that the seed oil profile of Camelina was amenable to modification and enhancement, we wished to further optimise the fatty acid composition via further iteration, and in doing so, also learn more about how the endogenous metabolic pathways are configured. We also wished to determine if environmental factors modulated the accumulation of native and non-native unsaturated fatty acids of Camelina seed oil. In addition, since the major application for any novel plant-derived sources of omega-3 LC-PUFAs is as a direct replacement for fish oil in aquafeed diets, it is important to reduce the level of omega-6 (n-6) fatty acids as much as is possible, since these are only present in low levels in marine organisms^20–22^. We therefore designed a new iteration to attempt to reduce the levels of omega-6 linoleic acid (LA; 18:2Δ^9,12^). This new line formed the experimental basis for querying all of the above, with a focus on how growth in the “real world” might impact on seed oil composition.

## Results

### Optimising the fatty acid profile of Camelina seed oil

**Figure 1:**
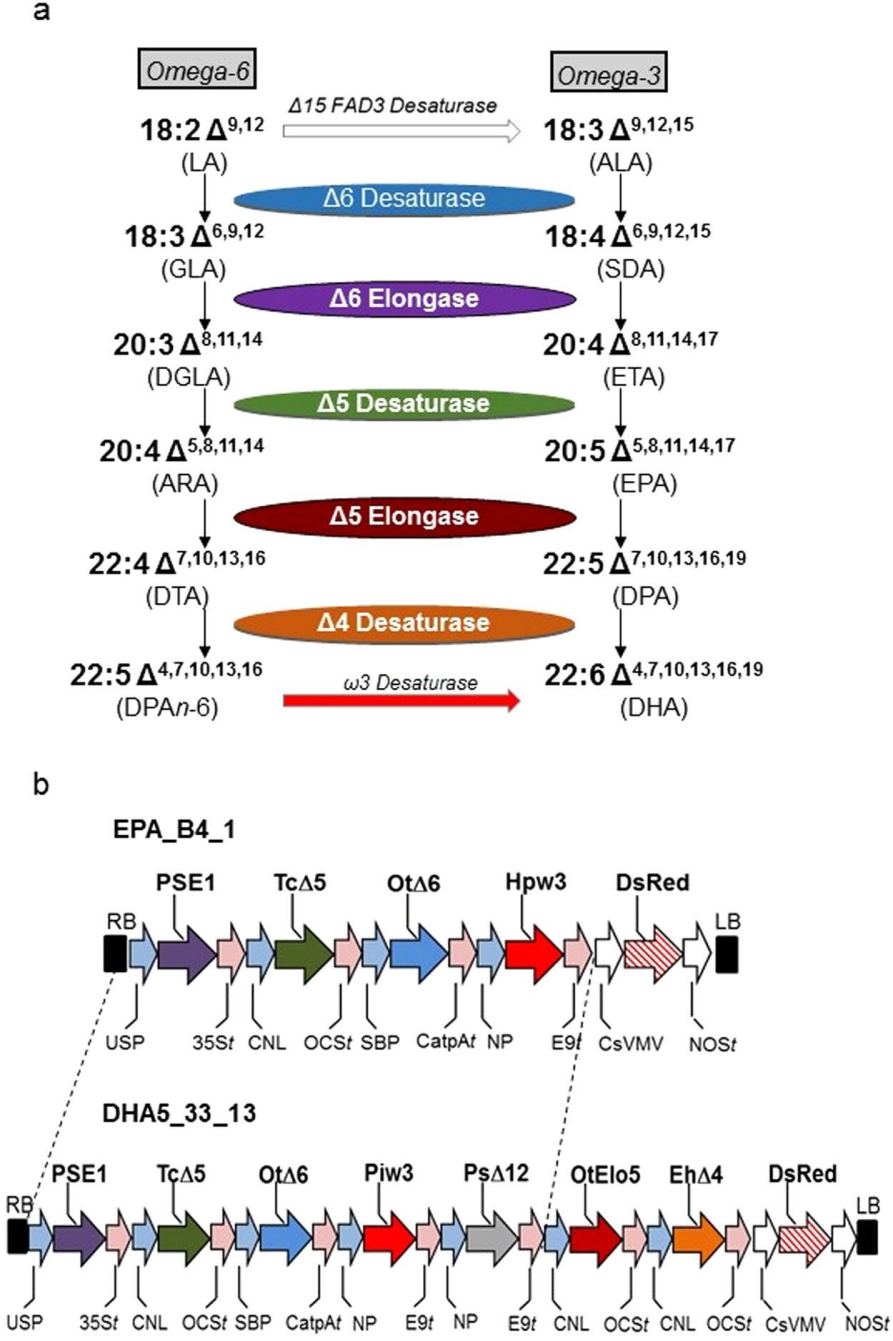
Production of EPA and DHA in the seeds of *C. sativa*. (**a**) The conventional Δ6 biosynthetic pathway for the production of omega-3 LC-PUFAs EPA and DHA, with the various desaturase and elongase enzyme activities shown in different colours [also used in (**b**)]. (**b**) Simplified maps of vectors EPA _B4_1 and DHA_33_13 used for transformation of *C. sativa*. Abbreviations: Cnl, conlinin 1 promoter for the gene encoding the flax 2S storage protein conlinin; USP, promoter region of the unknown seed protein of *Vicia faba*; SBP, sucrose binding protein 1800 promoter; NP, napin; Ot∆6, ∆6-desaturase from *O. tauri*; Tc∆5, a Δ5-desaturase from *Thraustochytrium* sp.; Piw3, ω3-desaturase from *Phytophthora infestans*; Ps∆12, a Δ12-desaturase from *Phytophthora sojae*; EhΔ4, ∆4-desaturase from *E. huxleyi*; PSE1, a Δ6-elongase from *P. patens*; OtElo5, Δ5-elongase from *O. tauri*; OCS, 35S, E9 and CatpA represent terminators.

The accumulation of oleic acid (OA; 18:1 Δ^9^) has been observed to decrease significantly in transgenic Camelina plants accumulating EPA and/or DHA^13^, even though OA is not a direct substrate for any of the transgene-derived enzymes required for the synthesis of omega-3 long chain polyunsaturated fatty acids. Conversely, the accumulation of LA was seen to remain constant, despite LA being a substrate for the transgene-derived Δ6-desaturase activity used in this study (Fig. 1)^23^. This is exemplified by the seed oil profile reported by Ruiz-Lopez *et al*.^13^, of transgenic Camelina line A5.1, in which high levels (24%) of EPA accumulated, with similar levels of LA (19%), but low levels of OA (3%). In wildtype (WT) Camelina, the endogenous levels of these two endogenous fatty acids are 18% LA and 13% OA, respectively^13^. One possible reason for the reduction in OA and maintenance of LA levels in the A5.1 transgenic lines was the presence of a Δ12-desaturase from the oomycete *Phytophthora sojae*, which is predicted to convert OA to LA, thus providing additional substrate for the *Ostreoccocus tauri* Δ6-desaturase present in the same expression cassette^12, 13^. Alternatively, the decrease in OA could result from a strong flux resulting from the depletion of LA via the action of the *O. tauri* Δ6-desaturase. To eliminate the first possibility, we designed a construct (designated EPA-B4.1), in which the *P. sojae* Δ12-desaturase was removed. Simultaneously, we replaced the ω3-desaturase from *Phytophthora infestans* ^24^ with an equivalent activity from *Hyaloperonospora parasitica*, the difference being that the *H. parasitica* ω-3 desaturase has a wider substrate-specificity, utilising both C18 and C20 omega-6 fatty acids^25^. Thus, EPA-B4.1 contained only four omega-3 LC-PUFA biosynthetic activities (Fig. 1a,b) and also differed in the use of DsRed as the (visible) marker for T-DNA transfer, as opposed to *nptII*-derived kanamycin-resistance previously used in A5.1^13^. In all other respects, the two constructs A5.1 and B4.1 were identical, and shared the other omega-3 LC-PUFA biosynthetic activities and regulatory elements. Transgenic Camelina lines were generated by *Agrobacterium*-mediated floral dip as previously described^13^, with primary transgenic seeds being identified by screening for the fluorescence of the DsRed protein. These seeds were grown up, and individual lines selfed towards homozygousity. Analysis of seed fatty acid methyl esters from glasshouse (GH) grown T3 plants indicated the accumulation of significant levels of EPA (~17% total fatty acids), confirming the efficient functioning of the heterologous omega-3 LC-PUFA biosynthetic machinery. Closer examination of the seed fatty acid methyl esters (FAMEs) from GH-grown T4 plants revealed that OA levels were restored to levels similar to that observed in WT seed, whereas the levels of LA were reduced by approximately a third (from 18% to 12%). On the basis of these data, it was concluded that EPA-B4.1 lines represented interesting experimental material for further evaluation at the field scale.

### Field evaluation of GM Camelina accumulating omega-3 LC-PUFAs

Seeds for line B4.1 were sown according to the conditions described in the 14/R8/01 DEFRA Consent for experimental environmental release (described in ref. 19), using the plot layout described in Supp. Fig. S1. In addition to control non-GM Camelina, we also included the line DHA5_33_13, which accumulates moderate levels of both EPA and DHA, and whose field performance for the 2014 season was recently reported^19^. In this way, the EPA-accumulating line B4.1 was not only compared with the performance of non-transgenic Camelina, but also against the benchmark of earlier iterations. It is also relevant to note that DHA5_33_13 also contained the Δ12-desaturase from *P. sojae* (Fig. 1b) and therefore displayed a seed fatty acid profile in which OA levels were reduced, but LA levels were unchanged, analogous to the EPA_A5.1 iteration described above. Thus, we hoped to be better able to determine the contribution of this particular desaturase to the accumulation of target fatty acids under field conditions.

Triplicated 10 m × 1.8 m strips were replicated in two blocks, meaning that for each line (EPA_B4.1, DHA5_33_13, WT) a total area of 108 m^2^ was sown, at seed rates judged to give stand densities compliant with the Consent (i.e. up to 300 plants/m2). Each strip was sown, maintained and harvested independently (see Supp. Fig. S1 for more details). GM and non-GM seeds were drilled on the 14^th^ April 2015, as was the 12m-wide pollen barrier, which also comprised of non-GM Camelina. However, this non-GM Camelina pollen barrier was sown at a higher seed rate and therefore not included in the analysis reported here. The experimental plots were subsequently top-dressed with nitrogen fertilizer at a rate of 370 kg/ha, and the established crop was treated with a graminicide grass-specific herbicide, but no additional inputs were applied apart from occasional irrigation. The two GM lines EPA_B4.1 and DHA5_33_13 grew at a similar rate to the WT control, and displayed no obvious morphological or phenotypic difference, in agreement with our observations of GH-grown material. The experimental plot and their component strips were harvested on the 9^th^ Sept 2015 using a plot combine. Seeds were threshed, cleaned and subject to further evaluation.

### Fatty acid composition of field grown events

**Figure 2:**
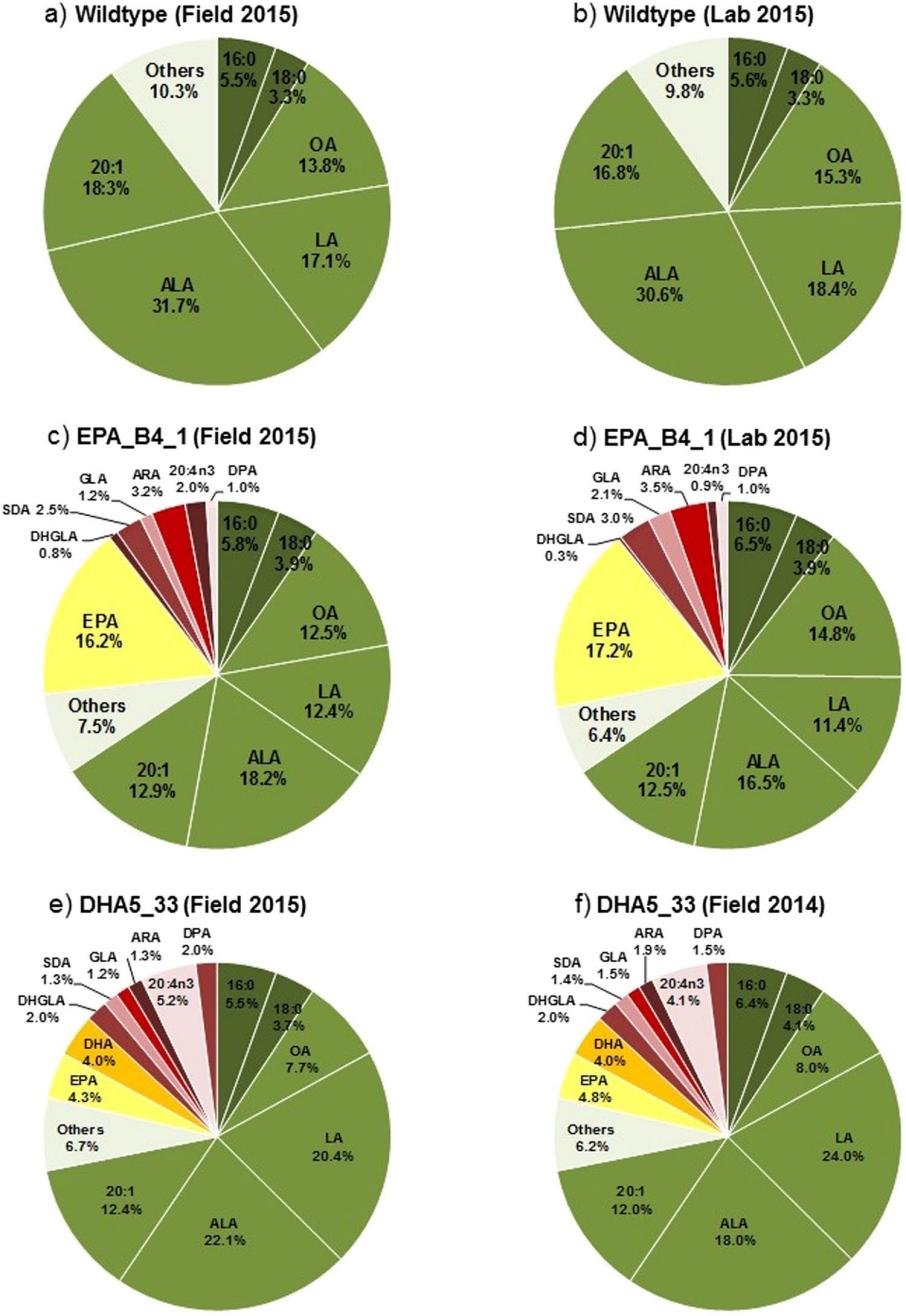
Total fatty acid composition (mol%) of laboratory and field-grown wild-type and engineered *C. sativa* seeds. Distribution of FAMEs in wildtype *C. sativa* grown (2015) in the field (**a**) and laboratory (**b**); the EPA producing line EPA_B4_1 grown (2015) in the field (**c**) and laboratory (**d**); and a comparison of the DHA producing line, DHA5_33, grown in the field in 2014 (**e**) and 2015 (**f**). An average of 75 seeds analysed by GC-FID (flame ionisation detection). Endogenous fatty acids are shown in shades of green; intermediates of the introduced biosynthetic pathway are shown in shades of red, and the key target fatty acids (EPA and DHA) are shown in shades of yellow.

The fatty acid composition of field grown Camelina seeds was determined by FAMes after subsampling of pooled seeds from individual strips (EPA_B4.1*2A, EPA_B4.1*2B etc.; see Supp Fig. S1 for more details). In Fig. 2, the total seed fatty acid composition is shown for the EPA-accumulating line (EPA_B4.1; Panels c, d) and the EPA/DHA-accumulating line (DHA5_33_13; Panels e, f), in both cases compared against the field-grown WT control fatty acid profile (Panels a, b). As is obvious, a number of non-native fatty acids are present in the transgenic lines (Fig. 2c–f) which are otherwise absent in the WT (Fig. 2a,b). In the case of EPA_B4.1, the most abundant new peak was identified (on the basis of retention time and co-migration with authentic standards) as being EPA (Fig. 2c) – this was also confirmed by mass spectrometry. The mean accumulation of EPA in mature field-grown Camelina was 16% of total seed fatty acids. In addition to the accumulation of EPA, the C20 LC-PUFAs eicosatetraenoic acid (ω-3 ETA, 20:4 Δ ^8,11,14,17^) and arachidonic acid (ω-6 ARA, 20:4 Δ^5,8,11,14^) accumulated to 2% and 3%, respectively, and the non-native C18 PUFAs, stearidonic acid (ω-3 SDA, 18:4Δ ^6,9,12,15^) and γ-linolenic acid (ω-6 GLA, 18:3 Δ^6,9,12^) accumulated to 1.7% and 1% respectively (Fig. 2c). As well as the substantial accumulation of these non-native fatty acids, there was also change in the profile of the endogenous fatty acids, compared with WT Camelina seeds. In particular, α–linolenic (ALA; 18:3 Δ ^9,12,15^) was significantly decreased (from 32.3% to 17.7%). Similarly, LA was reduced from 17.2% to 11.4% and 20:1 (n-9) was decreased from 17.6% to 11.6%. However, the levels of OA were essentially unchanged compared with WT, (12.1% versus 11.6%) (Fig. 2a–c). This latter observation was in line with the prediction that the absence of the *P. sojae* Δ12-desturase from the EPA_B4.1 construct would rebalance the levels of OA, though it is interesting to note that this was not the case for all other monounsaturated fatty acids (e.g. 20:1, ω-9) (see Discussion below). The total fatty acid profile of mature seeds from line DHA5_33_13 was similarly compared with WT, and seen to be in agreement with the data previously published on the field performance of this line^19^. In addition to the accumulation of omega-3 LC-PUFAs such as EPA, DPA and DHA to a combined level of ~15%, OA was reduced to 7% whereas LA was slightly increased to 21% (Fig. 2e). Since the DHA5_33_13 construct contained the *P. sojae* Δ12-desturase, which only recognises C16/18 substrates, it can be concluded that this enzyme activity was primarily responsible for modulating the ratio of OA and LA in the seeds of this transgenic Camelina line.

**Figure 3:**
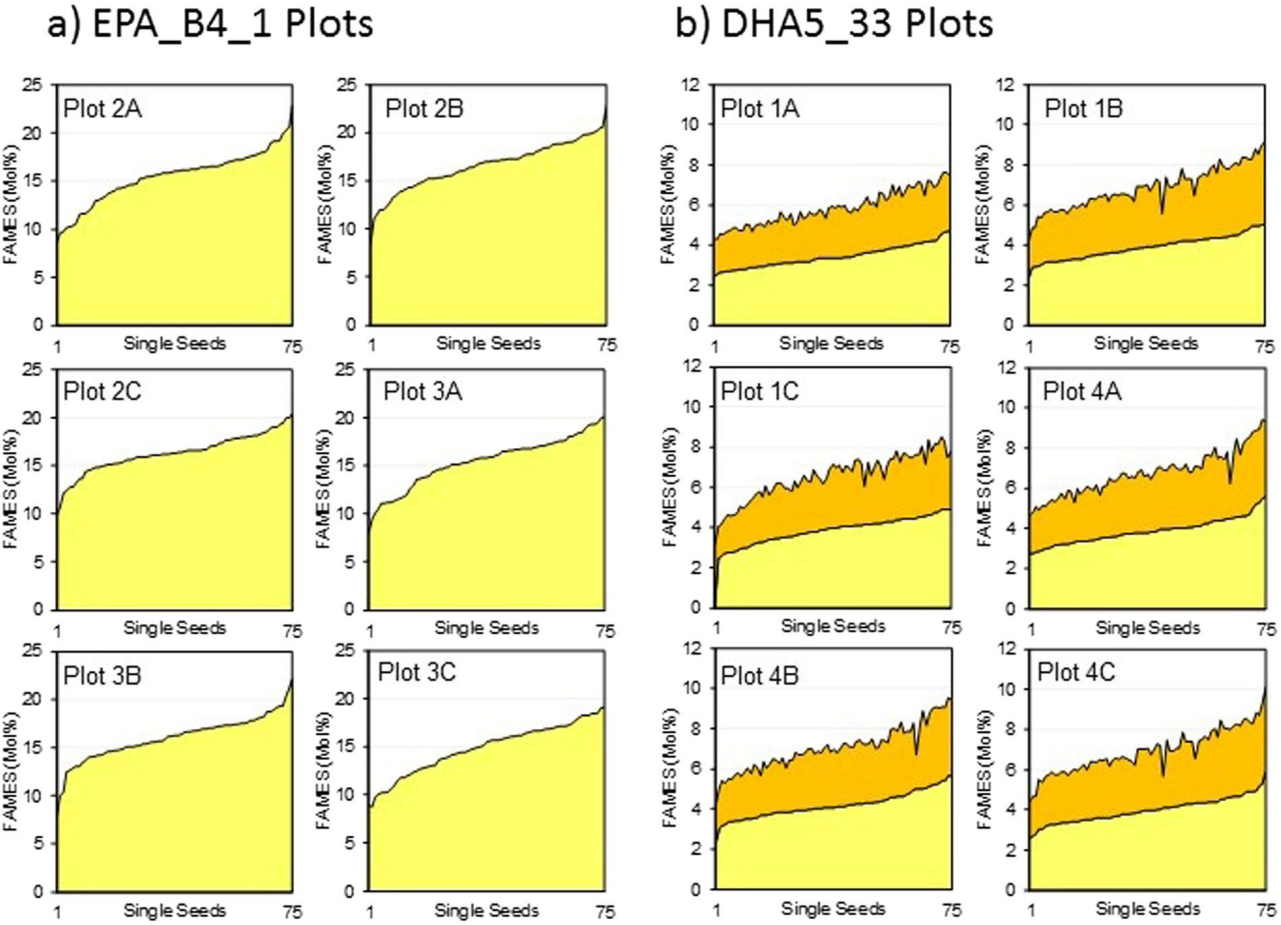
The accumulation of EPA and DHA in transformed field-grown seeds of *C. sativa*. The levels of specific n-3 EPA and DHA are shown in (**a**) and (**b**) for the individual sub plots of EPA_B4_1 and DHA5_33, respectively. The results are based on single seed analysis (n = 75); values for EPA are shown in yellow and DHA represented in orange.

To better determine the range of target fatty acids accumulated by these transgenic Camelina lines, FAMEs analysis of single seeds was carried out on 75 seeds per plot, generating the graphs shown in Fig. 3. In the case of EPA_4.1 (Fig. 3a), the minimum and maximum levels of EPA observed across all six plots were 8% and 23%, respectively, but with the majority of the samples (>90%) falling in the range of 15% ± 2%. Interestingly, as shown in Fig. 3a, there was clear variation between plots in the max/min accumulation of EPA, although the average value remained the same. A similar pattern was observed for DHA5_33_13 (Fig. 3b), in agreement with previous examination of the field performance of this line^19^. There was a strong correlation between the accumulation of EPA and that of DHA, as might be expected (Supp. Fig. S2), though this is not completely uniform. In the case of EPA_B4.1, the most significant correlation was an inverse association between ALA levels and those for EPA, implying that ALA is being metabolised to generate EPA. Despite this, the total omega-3 fatty acids (C18+ ω-3) present in seeds of either EPA_B4.1 (39.2%) or DHA5_33_13 (38.8%) were greater than that found in WT Camelina seeds (32%) (Fig. 2). However, the omega-6/omega-3 ratio differed between the two transgenic lines, with EPA_B4.1 having a ratio 1:2.2 in favour of omega-3, whereas DHA5_33_13 had a ratio of 1:1.5, similar to that observed in WT Camelina (1:1.64). This difference predominantly reflects the contribution of the Δ12-desaturase to the generation of omega-6 LA from OA in the DHA5_33_13 line.

### Agronomic Performance

**Figure 4:**
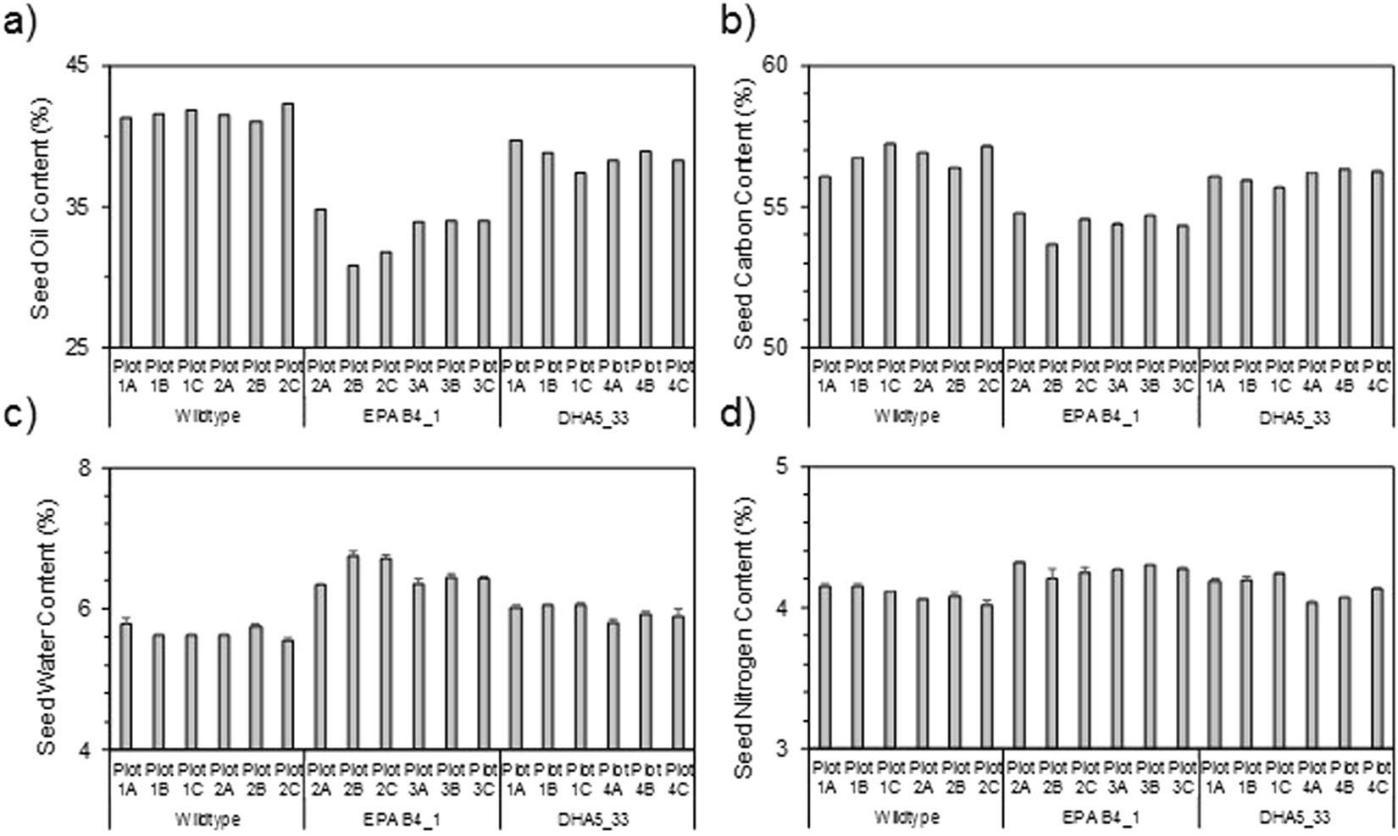
Seed yield and agronomic performance. (**a**) Total seed oil (n = 6+/− SE); (**b**) carbon (n = 6+/− SE); (**c**) water (n = 19+/− SE) and (**d**) nitrogen (n = 6+/− SE) content was determined and presented for each sub-plot of the field grown *C. sativa*.

Total seed lipid content was determined by the Soxhlet method. Approximately 100 g of seed from each plot was subject to this extraction protocol, and the results are shown in Fig. 4a. The average total seed lipid content for the non-GM WT Camelina was 41.6%. For the DHA5_33_13 line, the total seed lipid content was only marginally lower at 38.6%, whereas for EPA_B4.1, the total lipid content was reduced to 33.2%. Other parameters of the harvested seed were determined, including the total seed carbon and nitrogen (Fig. 4b,c) and also seed water content (Fig. 4d). Seed carbon was depressed in line EPA_B4.1, whereas the levels seen in WT and DHA5_33_13 were essentially the same – this is in agreement with the reduced oil content seen in the former line (Fig. 4a). Interestingly, both seed N and seed water content were elevated in EPA_B4.1, though these two parameters are unlikely to be directly linked with each other. Possible explanations for this variation are discussed below.

### Oil composition

**Figure 5:**
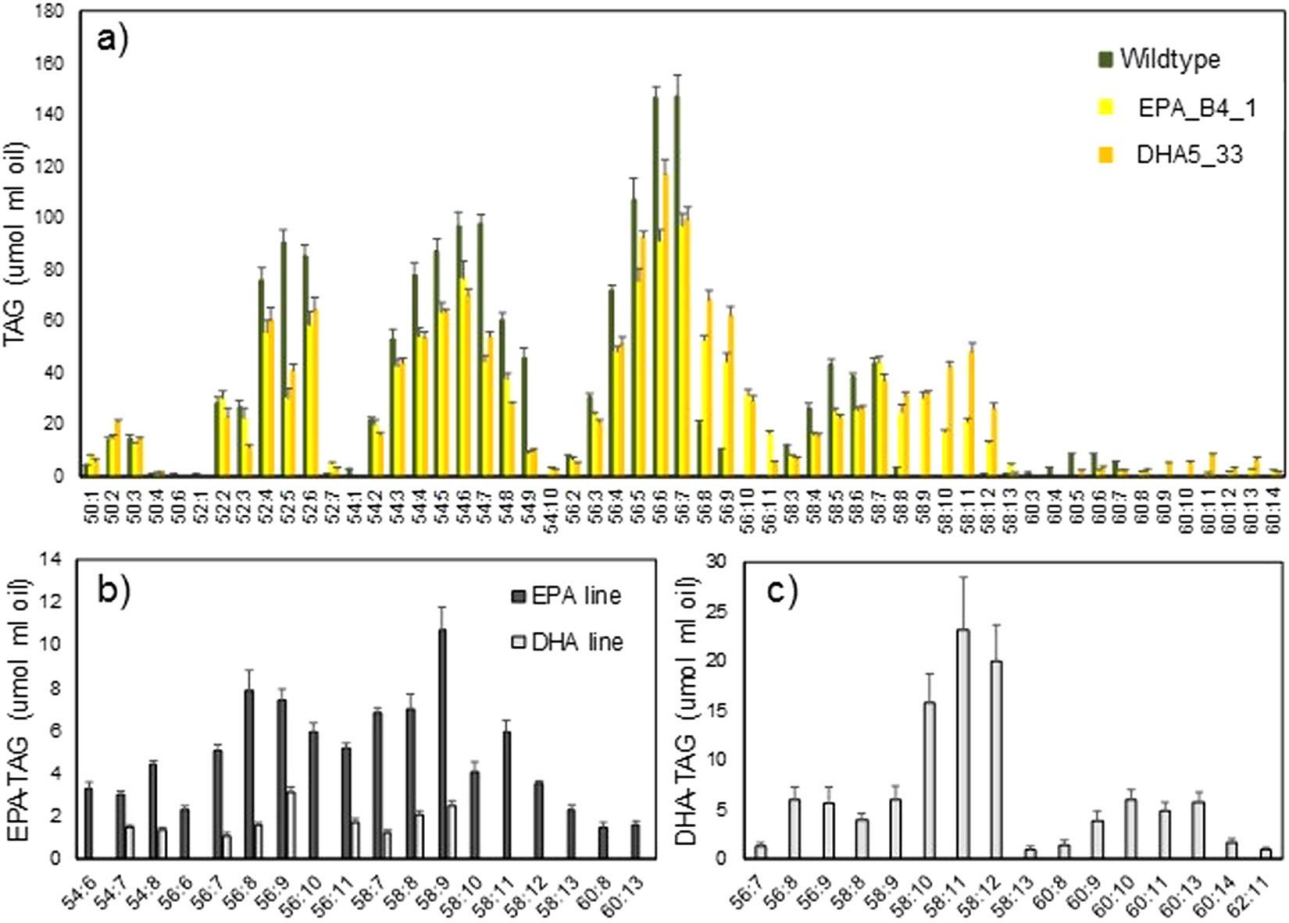
Analysis of triacylglycerols from mature *C. sativa* seeds. TAG molecular species were characterised from wild-type, EPA_B4_1 and DHA5_33 using a ESI-MS/MS neutral loss survey scan with each TAG species represented by the total number of fatty acid carbon atoms:desaturations. A Students T-test was used to independently compare *C. sativa* wildtype with EPA-B4_1 and DHA5_33. Those TAG molecular species with significantly (p 0.05) different accumulations are shown (**a**). Further analysis of the mass spectrometry data acquired for the engineered lines identified those significant TAG species containing either EPA (**b**) or DHA (**c**).

To more precisely define the oil composition of the different Camelina lines, the Soxhlet-extracted total seed lipid samples were analysed by a ESI-MS/MS fatty acid neutral loss survey scan to identify and quantify the individual triacylglycerol (TAG) molecular species present in the different plants. Using this method, it is possible to resolve TAG species on the basis of the mass to charge ratio (*m*/*z*), which is determined by the length of their constituent three fatty acids and the total level of unsaturation (number of double bonds). In this way, the abundance of specific TAG species comprised of different fatty acids can be estimated. As shown in Fig. 5a, WT Camelina seed oil contains a significant number of different TAG species, predominantly ranging from 52 to 58 carbons, and containing from two to up to nine double bonds. Although this method does not specifically identify the constituent fatty acids present in any given TAG species, in some cases only a particular combination can generate the detected *m*/*z*. For example, the moderately abundant TAG 54:9 present in WT samples is likely to be comprised of three ALA molecules attached to the glycerol backbone (since 18:3 + 18:3 + 18:3 = 54:9). The most abundant TAG species present in WT Camelina oil are 56:6 and 56:7, most likely comprised of 20:1, 18:3 and either 18:2 or 18:3, respectively. Notably, WT Camelina lacks any TAG species containing 10 or more double bonds. As can be seen in Fig. 5a, the TAG profile for both EPA_B4.1 and DHA5_33_13 is expanded, with the novel occurrence of additional C56/58 TAGs containing 10+ double bonds. Similarly, though to a lower level, highly polyunsaturated C54 and C60 TAG species were detected too in both transgenic lines which were absent in the WT control. It is important to note that these novel TAG species occur in addition to the cohort found in the WT line, thus expanding the repertoire present in the seeds, as opposed to reconfiguring the existing pool. In general, there were noticeable, but not major, changes in the TAG profiles for the two GM lines (Fig. 5a).

It is also possible to extend this technique to identify particular TAG species which contain fatty acids of interest. Thus, it was possible to identify TAG species which contained either EPA (Fig. 5b) or DHA (Fig. 5c). In the case of EPA-containing TAG species, the profiles differed between EPA_B4.1 and DHA5_33_13 (the latter which also accumulates EPA along with DHA). In the oil of EPA_B4.1, a number of C58 polyunsaturated TAGs were identified, not present in DHA5_33_13. These TAG species, 58:10–58:13 most likely represent TAG moieties containing two EPA molecules and a C18 acyl chain varying in saturation (from 18:0 to 18:3). The occurrence of these di-EPA TAG species most likely reflects abundance of EPA as a substrate for acylation present in EPA_B4.1, not mirrored in the reduced accumulation in DHA5_33_13. The most abundant EPA-containing TAG species in EPA_B4.1 were 58:9 (presumptively EPA, 20:1, 18:3), 56:9 (EPA, 18:3, 18:1/EPA, 18:2, 18:2) and 56:8 (EPA, 18:2, 18:1) with 58:9 and 56:9 being the most abundant in DHA5_33_13 (Fig. 5b). Interestingly, when the same TAG profiles are investigated for the presence of DHA (present only in DHA5_33_13), then the dominant species identified as containing that fatty acid are 58:10–58:12, with some significant accumulation of C60 species. In the case of the C58 TAG species, although it is possible that these could contain both EPA (20:5) and DHA (22:6) (e.g. 58:11 = 22:6, 20:5, 16:0), the failure to detect EPA (Fig. 5b) in such a TAG from DHA5_33_13 would rule this permutation out. We consider it more likely that 58:11 is comprised 22:6, 18:3, 18:2. Using similar reasoning it is possible to conclude that 60:13 contains both DHA and EPA, since scanning for either fatty acid generated a positive response (Fig. 5b,c) – thus, 60:13 most likely comprises 22:6, 20:5 and 18:2.

### Defining the location of novel fatty acids with mass spectrometry imaging

**Figure 6:**
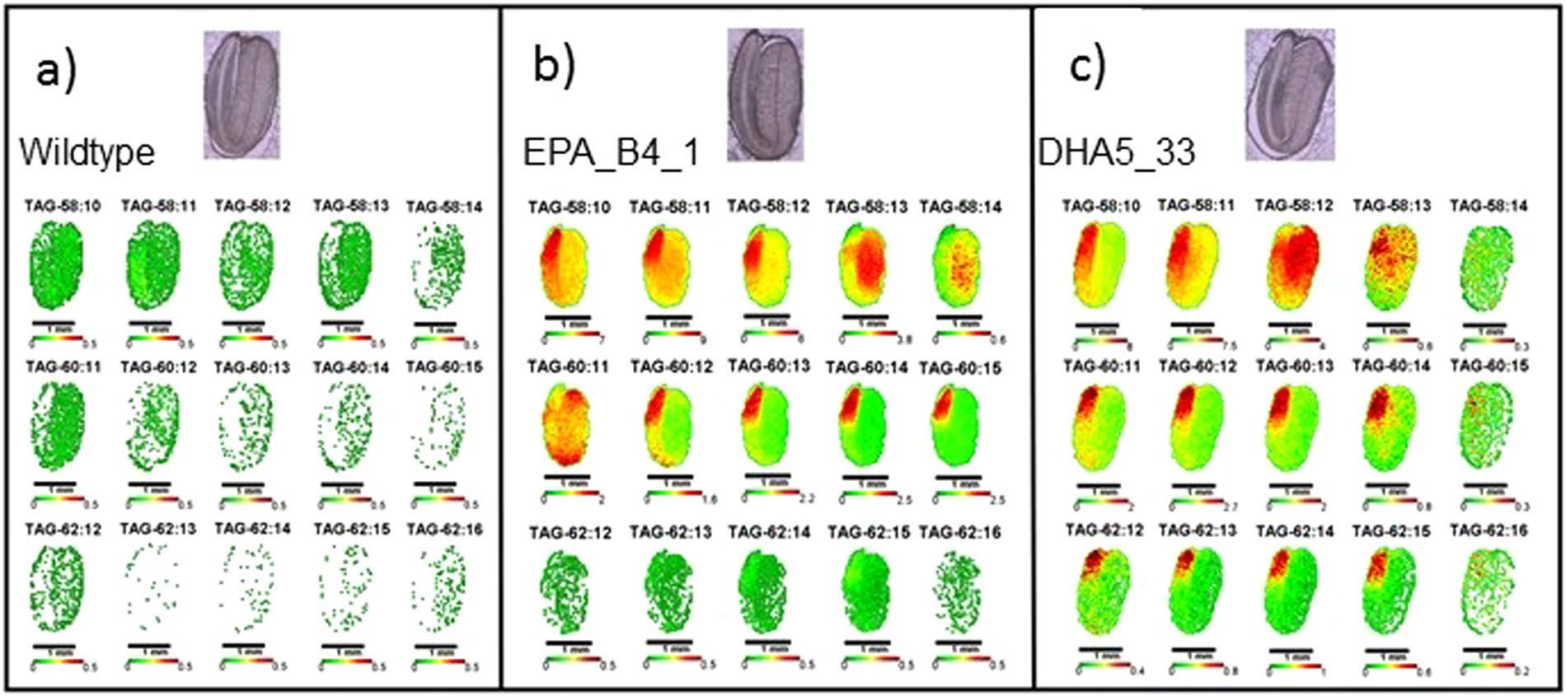
Representative images of TAG metabolites in mature field-grown seed of *C. sativa*. Cross-sections of embryos show the spatial distribution of specific TAG molecular species in wildtype (**a**), EPA_B4_1 (**b**) and DHA5_33_13 (**c**). Compare to bright-field images for orientation. False-colour images are converted from mol% of class amounts with *red* as the highest relative amount.

In addition to profiling the novel TAG species which resulted from the engineered accumulation of EPA and DHA, we wished to investigate the spatial distribution of these non-native fatty acids in the seeds of transgenic Camelina *in situ* by using matrix-assisted-laser-desorption-ionization (MALDI) coupled with high-resolution mass spectrometry (MS). By this method, it is possible to define (at ~40 micron scale) the location of the TAG species identified in Fig. 5a across a transverse section of a mature Camelina seed. Such sections were scanned for the presence of known TAGs (on the basis of *m*/*z*), and the resulting data processed to generate “heat-maps” reflecting their distribution and relative abundance. Presented in Fig. 6 (and part of a larger dataset) are the false-colour images of seed sections for TAG species which were present in the two GM lines and were predicted to contain either EPA or DHA (Fig. 5b,c; described above). A number of observations are clear from this analysis. Firstly, the distribution of different TAG species is not uniform across the seed, with obvious differences between the radicle and cotyledons. Secondly, in the case of the more unsaturated, C60 TAG species, their accumulation appears to be strongly restricted to the radicle tip. Finally, for some specific TAG species, there was a difference in their distribution pattern depending on which omega-3 LC-PUFA was present in the molecule. For example, 58:12 (which is completely absent in WT seeds – Fig. 5a) shows specific accumulation in the radicle for line B4.1 (Fig. 6a), whereas in DHA5_33_13, the accumulation of this TAG is more uniform or slightly enhanced in the cotyledons (Fig. 6c). As discussed above, the 58:12 TAG present in B4.1 is most likely comprised EPA, EPA, LA, whereas the TAG species of the same *m*/*z* from DHA5_33_13is predicted to comprise DHA, ALA, ALA. As another example, the TAG species 58:10 shows strong radicle-specific localisation in DHA5_33_13, whereas such a discrete pattern is less obvious in B4.1 (Fig. 6b,c). However, this again reflects the presence of multiple different TAGs with a *m*/*z* matching that of 58:10. In the EPA-accumulating line B4.1, this again most likely comprises EPA, EPA and 18:0, whereas for DHA5_33_13, where this TAG species has been shown to accumulate DHA (Fig. 5c), the likely composition is DHA, ALA, OA (or DHA, LA, LA). To confirm the validity of these observations, mature Camelina seeds were manually dissected to separate the radicle from the cotyledon, and the fatty acids of these two different tissue types analysed by both GC-FID and mass spectrometry. As shown in Supp. Fig. S3, these data support the non-uniform distribution of specific fatty acids, especially with the preferential accumulation of EPA and DHA in the radicle (Supp. Fig. S3D,E). Equally noteworthy is the asymmetric distribution of some endogenous fatty acids between the radicle and the cotyledon, especially for 16:0 and ALA (Supp. Fig. S3A).

We also examined the impact of different environmental conditions on the tissue-specific distribution of TAG species, comparing their localisation in seeds that were either grown in field or in the GH. As shown in Supp Fig. S4, WT seeds grown under either condition report a very similar distribution of TAG species, including several negative controls (i.e. TAG species which are known to be only present in the transgenic Camelina – Fig. 5). In the case of the DHA5_33_13, accumulation across the seed of six different TAG species is very similar for both field-grown (Supp Fig. S4A) and GH-grown (Supp Fig. S4B) plants, with a radicle-specific accumulation pattern for the non-native EPA/DHA accumulating TAGs. Interestingly, this consistency between different environments was not so apparent for such TAG species in B4.1, though (as discussed above), this may reflect conflation of signals from differing cohorts of TAGs with the same *m*/*z*, which in turn may be subject to some variation according to growth conditions. As such, this would be quite subtle in difference, since the overall fatty acid profile for GH or field grown material is essentially the same (cf. Fig. 2c,d).

## Discussion

The capacity to direct the synthesis of non-native fatty acids such as EPA and DHA in transgenic Camelina represents a significant achievement for plant biotechnology, not least of all given the importance these omega-3 fatty acids play in both aquatic and terrestrial food chains, including human nutrition^1–3^. However, several challenges remain, including the tailoring of the seed oil profile to be optimal for a given end-use, demonstrating the robustness of the omega-3 trait under field conditions, and trying to better define the biosynthetic route (at several levels – cellular, tissue-specific) to ultimately allow for a more predictive and precise manipulation of seed lipid metabolism^26^. In this study, we evaluated the possible reasons for the previously observed^13^ decrease in OA in transgenic Camelina, from the perspective of understanding this alteration and also tailoring the seed oil to be better matched with the needs of aquaculture. Based on the data presented here, the presence of an oomycete Δ12-desaturase as part of the transgenic pathway for the synthesis of omega-3 LC-PUFAs plays a major role in determining the ratio of OA to LA, but without significant impact on the accumulation of EPA. Specifically, this activity is responsible for the conversion of OA to LA, resulting in the depletion of total OA levels. In the case of the B4.1 construct, that lacks this Δ12-desaturase, OA levels were restored to WT levels, and the elevated levels of LA observed in both DHA5_33_13 and the previous EPA iteration A5.1 were reduced, to lower than WT. This may also imply that the LA derived from the oomycete Δ12-desaturase is somehow unavailable for further metabolism by the omega-3 pathway, explaining the apparent homeostasis of this fatty acid, despite the very significant depletion of the LA-catabolite ALA in all transgenic lines (cf. Fig. 2). Interestingly, Petrie *et al*.^27^ also reported the synthesis of EPA and DHA in transgenic Camelina, albeit with three different sets of heterologous genes. In that study, maximal levels of 12.4% DHA were reported for GH-grown material, though EPA levels were much lower (maximally 3.3% of total seed fatty acids). However, a more dramatic difference is seen in the levels reported for endogenous fatty acids, with no significant decrease in OA levels observed, but large decreases in both LA (from 18.1% to 8.4%) and ALA (from 38.2% to 29.4%)^27^. This is unlike the situation described here for either GH or field-grown material, where ALA, but not LA, appears to be the primary catabolite used for the synthesis of EPA and DHA. There are a number of differences between this study and that of Petrie *et al*.^27^, the most obvious being that the constructs used in the latter study all contained a Δ12-desaturase (from the yeast *Lanchancea kluyveri*). It is therefore surprising that the endogenous levels of OA are not impacted by the presence of that transgene, though it is difficult to assess its individual activity since the product (LA) is clearly efficiently metabolised by the transgene-encoded ω3-desaturase from *Pichia pastoris* used in that study. Thus, one scenario could be that the *L. kluyveri* Δ12-desaturase makes only a restricted contribution to the flux of fatty acids towards EPA and DHA. However, in the absence of a similar construct lacking the *L. kluyveri* Δ12-desaturase, analogous to B4.1 described in this study, the role of that enzyme remains open. Irrespective of that, it is clear from our data that the presence of a Δ12-desaturase is not a significant activity for the high level accumulation of EPA and DHA, and that the absence of such a transgene activity may be desirable in terms of optimising the omega-6/omega-3 ratio of seed oil. Of equal note, Petrie *et al*.^27^ obtained the highest levels of DHA from a construct (mod-F) which contained two copies of the Δ6-desaturase, although the contribution of this additional activity appears marginal when compared against the levels of DHA in the two constructs which contained only a single copy of this desaturase (GA7 = 9.6% ± 0.2; mod-G = 11.5% ± 0.8).

Regarding the performance of GM Camelina B4.1 under field conditions, it is clear that whilst the levels of EPA accumulated in seed oil were every similar for either GH or field conditions (Fig. 2c,d), other parameters showed some variation. In particular, seed oil content and total seed carbon were slightly depressed in field-grown plants, and showed an inverse correlation with an increase in seed water content and total seed nitrogen for B4.1. However, in the case of DHA5_33_13, there was no meaningful variation in seed oil content compared with the equivalent field-grown WT, nor for total seed carbon or seed nitrogen. This was in agreement with our previous assessment of the agronomic field performance of this line^19^, as was our observation that seed water content was slightly elevated in DHA5_33_13 (though not as high as for B4.1). Some aspects of these observations for B4.1 can be rationalised, such as the reduced total seed carbon is a direct reflection of the reduction in total seed oil content, and that as a consequential, compensatory action, total seed nitrogen is increased (via elevated seed protein accumulation). Less obvious is the increased seed water content, which might be inversely related to seed oil content, though mechanistically this is hard to explain. The reduction in total seed oil content in B4.1 was surprising, not least of all since such an oil-yield penalty phenotype was not previously observed in GM Camelina accumulating EPA and DHA^14^ nor in our current field trial of that same line. In comparison, Mansour *et al*.^28^ reported that the seed oil content of GH-grown DHA-accumulating Camelina was slightly reduced compared to WT (36% versus 41%), though the authors noted that this observation would need additional replication to determine if this was statistically valid. Of similar relevance is the recent report on canola plants accumulating low levels of EPA and DHA (<4% of total seed oil) in which no oil yield penalty was observed, either from GH or field grown plants^29^. Further evaluation of different events derived from the B4.1 construct may also clarify any relationship between omega-3 content and seed oil content.

One novel aspect of this study is through the use of MALDI-MS^30^ imaging to examine the discrete accumulation of different fatty acids in the Camelina seed, allowing us to identify and validate the preferential accumulation of EPA and DHA in the radicle compared to the more abundant cotyledon cells. Confirmation of this non-uniform distribution of fatty acids was provided by manual dissection of radicle and cotyledon tissue samples, followed by either GC-FID or ESI-MS/MS, and revealed that both native and non-native fatty acids have asymmetric accumulation- in the former case this was also observed in WT non-transgenic seeds and thus independent of genetic transformation. The MALDI-MSI approach was used (in conjunction with the detailed ESI-MS/MS definition of the seed TAG species in the different lines) to not only provide a tissue-specific digital “fingerprint” for the accumulation of every possible TAG molecule (and their constituent fatty acids), but also to determine if this very comprehensive dataset changed as a consequence of environmental conditions. Interestingly, there were only minor qualitative differences between the GH and field-grown material, at least as far as TAG species were concerned. From a different perspective, it will also be important to determine if the tissue-specific localisation of some particular lipids and their constituent fatty acids act as rate-limiting factors in the successful metabolic engineering of plant seeds for modified oil profile. It is unlikely that the asymmetric tissue distribution of EPA and DHA here is due to promoters differentially controlling transgene expression, since others have shown that the napin promoter is active uniformly throughout the embryo in maturing seeds^31^. Nevertheless, tissue-specific promoter activity remains a possibility that should be considered in metabolic engineering strategies. Perhaps more intriguing is the possibility that endogenous tissue-specific differences in enzyme distribution, inherent to Camelina embryos, may influence the accumulation of EPA and DHA in radicle tissues versus cotyledons tissues. Others have shown that the expression and suppression of different acyltransferases can influence the distribution of TAG species in Camelina seeds^32^, supporting earlier suggestions that a DGAT-dominated pathway may be favoured in cotyledons, while a PDAT-dominated pathway may be more prevalent in embryonic axis tissues^33^. Going forward, more precise cell type-specific expression of enzymes to optimise the flux of fatty acids towards the synthesis of EPA and DHA may need to be considered. Such future investigations will hopefully provide new insights into the compartmentalisation and regulation of plant lipid metabolism.

## Methods

### Plant material and growth conditions

*Camelina sativa* grown in the glasshouse were maintained in controlled conditions at 25 °C day/16 °C night, 50–60% humidity, and kept under a 16 h photo period (long day), with supplemental light provided when ambient levels fell below 400 μmol m^2^ s^1^. Harvest usually occurred 95 days after sowing.

### Generation of transgenic plants

Transgenic *C. sativa* lines were generated as previously described^13^. The designed vectors were transferred into *Agrobacterium tumefaciens* strain AGL1. *C. sativa* inflorescences were immersed in the *Agrobacterium* suspension for 30 s without applying any vacuum. Transgenic seeds expressing the EPA or EPA/DHA pathway were identified by visual screening for DsRed activity. Seeds harvested from transformed plants were illuminated using a green LED light. Fluorescent seeds were visualised using a red-lens filter.

### Vector construction

Two constructs, as described, containing cassettes of four (EPA_B4_1) or seven genes (DHA5_33_13; Fig. 1b) were used for plant transformation. The five-gene construct, contained an optimal set of genes for EPA synthesis: a Δ6-desaturase gene from *O. tauri* (OtΔ6^34^), a Δ6 fatty acid elongase gene from *Physcomitrella patens* (PSE1^35^,) a Δ5-desaturase gene from *Thraustochytrium* sp. (TcΔ5^36^), a Δ12-desaturase gene from *Phytophthora sojae* (PsΔ12^25^) and an ω3-desaturase from *Phytophthora infestans* (Pi-w-3^24^; or *Hyaloperonospora parasitica*, (Hp-ω3^25^). The *H. parasitica* ω-3 desaturase has a wider substrate-specificity, utilising both C18 and C20 omega-6 fatty acids. All genes were individually cloned under the control of seed-specific promoters, and then combined into a single T-DNA transformation vector as previously described^12^. All open reading frames for desaturases and elongases were re-synthesized (GenScript Corporation, NJ, www.genscript.com) and codon-optimized for expression in *C. sativa*. To create the seven-gene construct for EPA and DHA synthesis, OtElo5, an *O. tauri* Δ5 fatty acid elongase gene^37^, and EhΔ4, a Δ4-desaturase gene from *Emiliania huxleyi* ^38^, both flanked by conlinin promoters^24^ and OCS terminators, were added to the EPA_B4_1 construct. The destination vector contained a DsRed marker for visual selection via seed coat-specific expression of DsRed.

### Field trial

Field experiments were conducted at Rothamsted Research (Harpenden, Hertfordshire, U.K.; grid reference TL120130; Supplementary Fig. S1). The field trial site consisted of twelve 18 m^2^ sub plots of GM *C. sativa* and six 18 m^2^ plots of WT *C. sativa*, each separated by a 0.5 m unsown break. A total of four plots were established - duplicates of iteration A and C, each containing 3 subplots. The experiment was surrounded by a 12m-wide WT *C. sativa* strip which served as a “buffer” to mitigate the dispersal of GM pollen. *C. sativa* is a self-pollinating species, with very low rates of outcrossing or cross-pollination^39^. The trial plot was sown on the 14^th^ of Apr 2015, with T6 GM *C. sativa* seeds sown to create a standing plant density of 290/m^2^ and 300/m^2^ for the WT. Seedlings were irrigated as necessary following emergence and an insect-proof net was erected around the central experimental plot prior to flowering in order to prevent insect-mediated pollen dispersal; (net removed following the cessation of flowering). Plants were allowed to set seed and monitored for seed maturation. Both WT and GM *C. sativa* were harvested on the 9^th^ of September 2015 (148 day growing season) using a small plot combine, when seeds were fully mature and following three consecutive dry days. In order to complete the harvest, the pollen barrier was removed. The harvested seed was transported to the GM facility glasshouse to further dry before threshing using a HaldrupLT-20 laboratory thresher. Cleaned seeds were tested for DsRed activity, stored, double bagged in paper bags inside locking plastic boxes prior to analysis.

### Assessment of agronomic performance

Total carbon and nitrogen were determined by combustion using a Combustion Analyser (LECO TruMac, LecoCorp, St.Paul, MN). This was performed by the in-house analytical unit at Rothamsted Research. Data is present as a percentage of 100% dry matter content. Seed water content was determined from field grown WT, EPA-B4_1 and DHA5_33_13 sub plots. Three replicates of 1 g for each sub-plot were dried in an oven (80 ^o^C) until no further weight change could be recorded.

### Fatty acid analysis

Total fatty acids in seed batches were extracted and transmethylated according to the method of ref. 40. Methyl ester derivatives of total fatty acids extracted were analysed by Gas Chromatography-FID (flame ionisation detection) and the results were confirmed by GC–MS^12^. Values presented are from the analysis of single seeds.

### Lipid Analysis

For the validation of MALDI imaging, mature seeds of *C. sativa* were dissected, lipids extracted and analysed by ESI-MS/MS using methods adapted from Lee *et al*.^41^. Prior to lipid extraction, mature seeds (five replicates of 40 seeds for WT, EPA and EPA/DHA lines) were imbibed for 20 min and dissected manually to separate the embryonic axis and cotyledonary tissue. To extract lipids from the isolated radicle and cotyledonary tissue, the radicle and cotyledonary tissue for each replicate was ground to a powder and transferred immediately to 1 mL of hot (85 °C) isopropanol for 10 min. The homogenate was centrifuged at 300 *g* for 15 min at room temperature, supernatant was collected, and the pellet was re-extracted with isopropanol/chloroform (1:1 v/v) and washed with 1 mL of 1 M KCl and then 2 mL water. The solvent was evaporated under nitrogen, and the dry lipid extract was dissolved in 1 mL of chloroform. The molecular species of phosphatidylcholine (PC) were analysed by ESI triple-quadrupole mass spectrometry (4000 QTRAP; SCIEX). The molecular species of PC were defined by the presence of a head-group fragment (184 *m*/*z*) and the mass/charge of the intact lipid ion formed by ESI. Such tandem ESI-MS/MS precursor and product ion scanning, based on head-group fragment, do not determine the individual fatty acyl species. Instead, PC molecular species were identified at the level of class, total acyl carbons and total number of acyl carbon–carbon double bonds. Polar lipids were normalized by comparing PC internal standards (PC 28:0 and PC 48:0; Avanti Polar lipids) and expressed as a total percentage of the MS peak area signal.

Triacylglycerols (TAGs) were measured in both lipids extracted from dissected Camelina seed and cold pressed oil from seed harvested from the field trial. TAGs were measured according to ref. 42 and were defined by the presence of one acyl fragment and the mass/charge of the ion formed from the intact lipid (neutral loss profiling). This allows identification of one TAG acyl species and the total acyl carbons and total number of acyl double bonds in the other two chains. The procedure does not allow identification of the other two fatty acids individually nor the positions (sn-1, sn-2, or sn-3) that individual acyl chains occupy on the glycerol. TAGs were quantified after background subtraction, smoothing, integration, isotope deconvolution and comparison of sample peaks with those of the internal standard (using Lipid-View^TM^; Sciex). The profiling samples were prepared by combing 50 uL of the total lipid extract with 950 uL of isopropanol/methanol/50 mM ammonium acetate/dichloromethane (4:3:2:1). Samples were infused at 15 uL/min with an autosampler (CTC- PAL, CTC Analytics). The scan speed was 100 u/s. The collision energy, with nitrogen in the collision cell, was +25 V; declustering potential was +100 V; entrance potential was 14 V; and exit potential was +14 V. Sixty continuum scans were averaged in the multiple channel analyser mode. For product ion analysis, the first quadrupole mass spectrometer (Q1) was set to select the TAG mass and Q3 for the detection of fragments fragmented by collision induced dissociation. The mass spectral responses of various TAG species are variable, owing to differential ionization of individual molecular TAG species. For all analyses, gas pressure was set on ‘low’, and the mass analysers were adjusted to a resolution of 0.7 l full width height. The source temperature was 100 °C; the interface heater was on, and +5.5 kV was applied to the electrospray capillary; the curtain gas was set at 20 (arbitrary units; and the two ion source gases were set at 45 (arbitrary units). In the data shown herein, no response corrections were applied to the data. The data were normalized to the internal standards tri15:0 and tri19:0 (Nu-Chek Prep, Elysian, MN). A Students T-test (p value 0.05) was used to independently compare *C. sativa* wildtype with EPA-B4_1 and DHA5_33.

Spatial distributions of TAG were determined *in situ* by matrix-assisted laser desorption/ionization mass spectrometry imaging (MALDI-MSI). Mature *C. sativa* seed were embedded in porcine gelatin and then frozen to −80 °C, before equilibration to −20 °C. Tissues were cut into 40-μm-thick cross sections on a cryostat (Leica CM1950) at −18 °C and lyophilized overnight before application of matrix (2,5-dihydroxybenzoic acid) by sublimation. A MALDI Orbitrap hybrid mass spectrometer (MALDI LTQ Orbitrap-XL; Thermo Fisher Scientific, Waltham, MA, USA) was used to scan the tissue sections at 40 micron steps, and the MS data were used to reconstruct false-colour MALDI-MSI images based on ion counts of selected lipid species^31^. Lipid species were identified by exact mass comparisons with the LIPID MAPS database (http://www.lipidmaps.org/), and confirmed by MS/MS. Detailed procedures for data acquisition and analyses were described previously^30^.

## Electronic supplementary material

Supplementary Information
