# Supplementary material for: Tailoring seed oil composition in the real world: optimising omega-3 long chain polyunsaturated fatty acid accumulation in transgenic Camelina sativa
Source: Sci Rep. 2017 Jul 26;7:6570. doi: 10.1038/s41598-017-06838-0 (PMC5529437; doi:10.1038/s41598-017-06838-0)
Supplement: Supplementary file 1 — Supplementary Information [file 41598_2017_6838_MOESM1_ESM.pdf]

Supplementary Data

Tailoring seed oil composition in the real world: optimising omega-3 long chain polyunsaturated fatty acid accumulation in transgenic *Camelina sativa*

Sarah Usher, Lihua Han, Richard P. Haslam, Louise V. Michaelson, Drew Sturtevant, Mina Aziz*, Kent D. Chapman, Olga Sayanova, Johnathan A. Napier*

Author for correspondence - johnathan.napier@rothamsted.ac.uk

Supplementary Figure S1

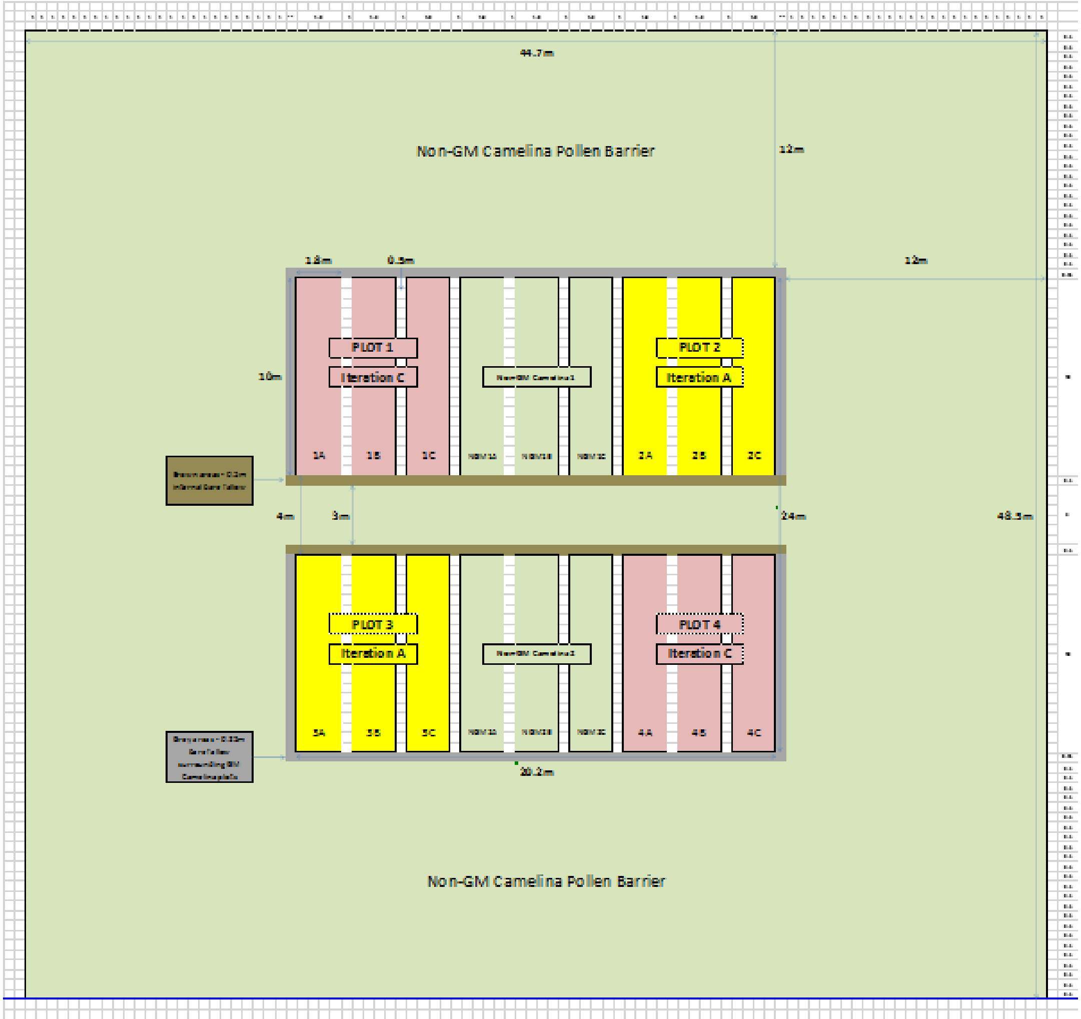

Supplementary Figure S1. Location and plot description of the *C sativa* trial on the Rothamsted Experimental farm. The non-GM pollen barrier (12m) surrounded the individual trial sub-plots (1.8 x 10m). Three central sub-plots were sown with wildtype *C. sativa*, whilst EPA_B4_1 (annotated as Iteration C) and DHA5_33 (annotated as Iteration A) were sown in plots 1 and 4 or 2 and 3 respectively.

See also https://www.gov.uk/government/publications/genetically-modified-organisms-rothamsted-research-14r801 for additional details on plot layout.

Supplementary Figure S2

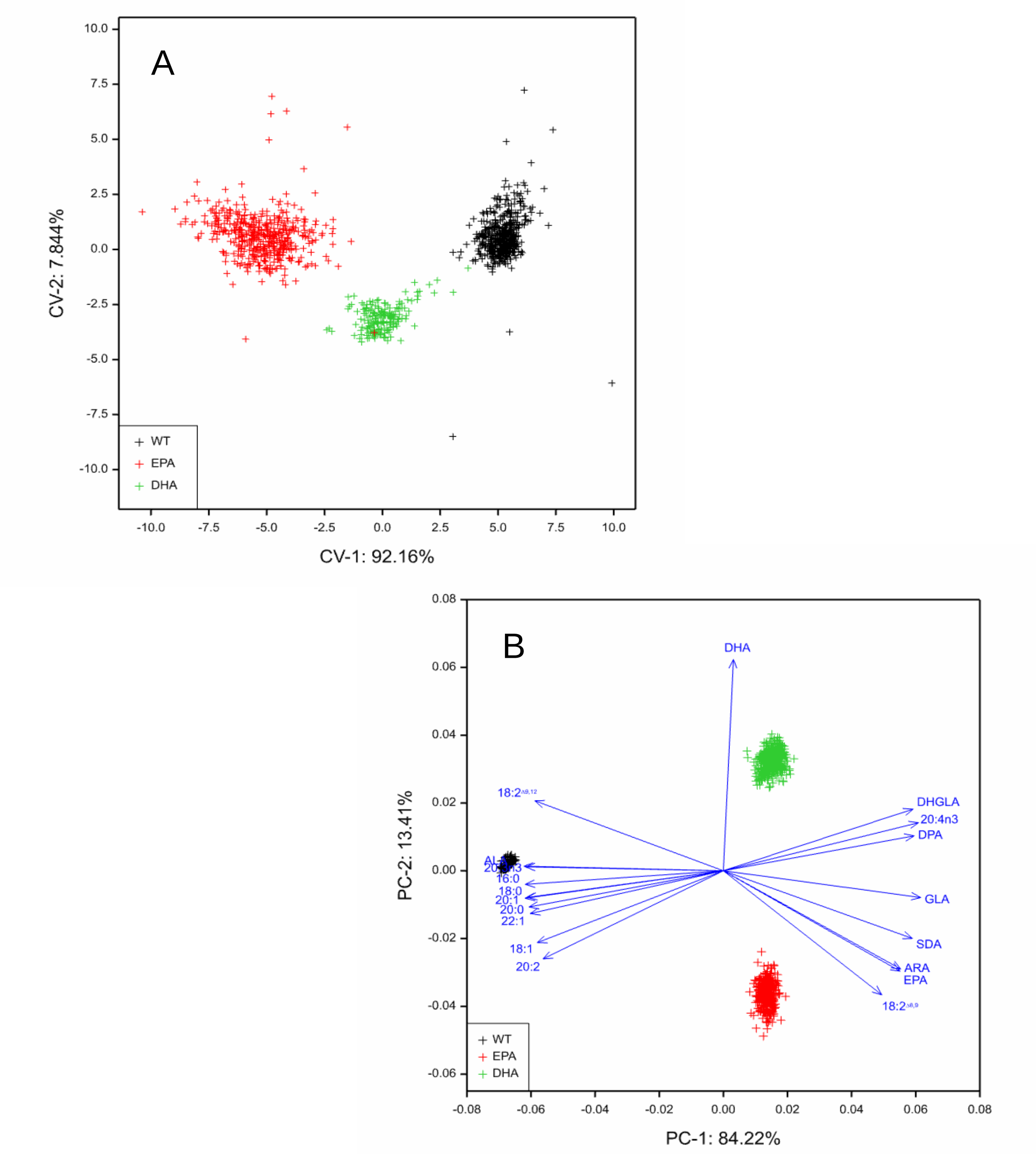

Supplementary Figure S2. Multivariate statistical analysis of the field trial *C.sativa* single-seed FAMEs data. Data consisted of compositional measurements of 21 fatty acids as a percentage of total abundance. These were collected from 1081 single seed samples across three different genotypes with a biological replication of two and a maximum of 226 technical replicates from each block. *Canonical Variates Analysis* (a). After transformation canonical variates analysis (CVA) was used to explore the multivariate response of the fatty acids (as a relative measure to the geometric mean of seed fatty acid abundance) in the different treatment conditions for the endogenous subset. Retaining all individual seeds allows all samples to be visualised in the CVA plot, however significance tests have not been performed due to the high level of pseudo-replication. *Principal Components Analysis* (b). After transformation principal components analysis (PCA) was used to explore the multivariate response of the fatty acids (as a relative measure to the geometric mean) in the different treatment conditions for the complete seed subset. Note that the presence of structural zeros violates the assumption of constant within group variance-covariance matrix required for CVA. PCA was calculated using the correlation matrix, to give equal importance to each variable. Retaining the first two principal components, that account for a substantial proportion of the total variation in the data, allowed the samples to be visualised and compared simply in a two-dimensional plot. Analyses were performed in GenStat (17th edition, VSN International Ltd, Hemel Hempstead, UK). From the scatterplot of the two canonical variates for the transformed endogeneous fatty acid dataset. It is clear, that in the first dimension (explaining the vast majority of the variation within the data at 92.16%) separates out the three different genotypes. In particular, it pulls apart the DHA line from the EPA line, with WT seeds lying in between. The loadings can be used to find which lipids (relative to the geometric mean of lipid abundance) contribute most to the CVs and consequently to the discrimination. Fatty acids - 16:0, 18:1, 18:2n6,9 - all have high loadings indicating they have greatest discriminatory capability. The second canonical variate, although only explaining 7.84% of the variation in the data, pulls apart the GM lines from the WT with the key endogenous lipids being ALA, 20:1, 16:0 and 18:0. There is some reasonable spread in these figures, indicating a reasonable amount of seed-to-seed variation

Supplementary Figure S3

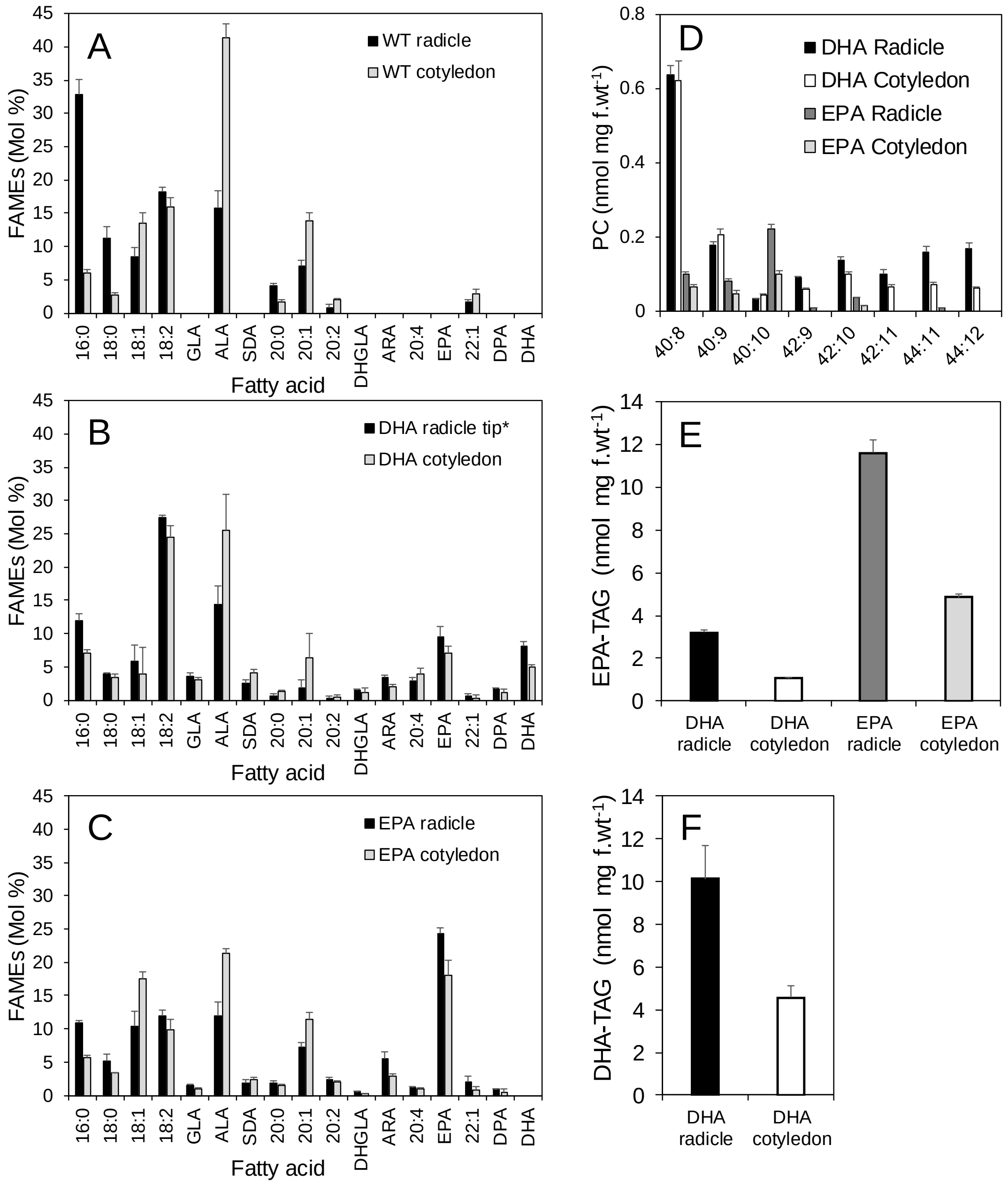

Supplementary Figure S3. Analysis of fatty acids and lipids from dissected mature *C. sativa* seed. To validate the heterogeneous distributions of PC and TAG in the mature seeds of engineered *C. sativa* seeds were manually dissected and the radicle and cotyledonary tissue isolated. Lipids were extracted from these tissues and transmethylated for GC-FID analysis (A-C) and lipidomics profiling i.e. PC molecular species (D) and TAG species contain EPA (E) and DHA (F). Note. * indicates that the data shown was specifically for the analysis of radicle tip tissue in the DHA5_33 line and not the entire embryonic axis. This reflected the localised distribution of PC 44:12 and TAG 60:12/13/14 at the radicle tip, as identified by MALDI-MS imaging. All values presented are the average of five replicate lipid extractions, +/- SE.

Supplementary Figure S4

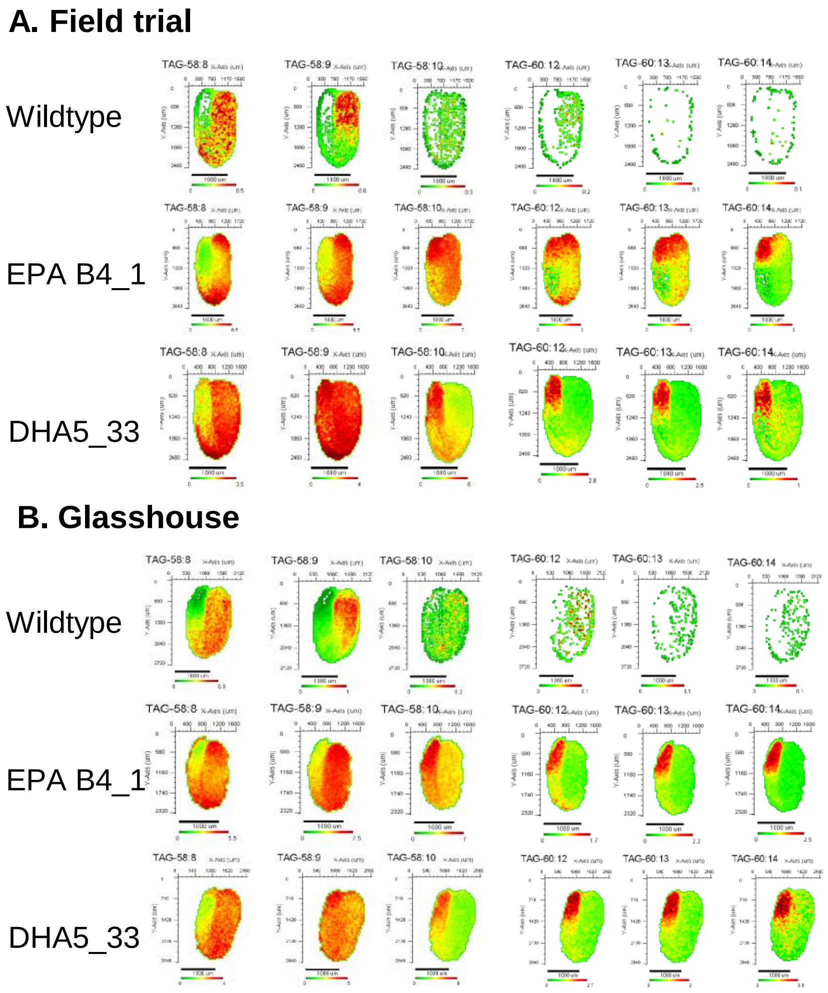

Supplementary Figure S4. A comparison of TAG distributions in field and laboratory grown mature *C. sativa* seed. Selected TAG molecular species (representative of a larger dataset) with asymmetrical seed distributions were used to make a comparison of field-grown (A) and glasshouse (B) material.
